# The effect of temperature on proliferation and differentiation of chicken skeletal muscle satellite cells isolated from different muscle types

**DOI:** 10.14814/phy2.12770

**Published:** 2016-04-28

**Authors:** Rachel L. Harding, Orna Halevy, Shlomo Yahav, Sandra G. Velleman

**Affiliations:** ^1^ Ohio Agricultural Research and Development CenterThe Ohio State UniversityWoosterOhio; ^2^ Department of Animal SciencesThe Hebrew University of JerusalemRehovotIsrael; ^3^ Institute of Animal SciencesAgricultural Research OrganizationThe Volcani CenterBet DaganIsrael

**Keywords:** Chicken, fiber type, muscle, satellite cells, temperature

## Abstract

Skeletal muscle satellite cells are a muscle stem cell population that mediate posthatch muscle growth and repair. Satellite cells respond differentially to environmental stimuli based upon their fiber‐type of origin. The objective of this study was to determine how temperatures below and above the in vitro control of 38°C affected the proliferation and differentiation of satellite cells isolated from the chicken anaerobic pectoralis major (p. major) or mixed fiber biceps femoris (b.femoris) muscles. The satellite cells isolated from the p. major muscle were more sensitive to both cold and hot temperatures compared to the b.femoris satellite cells during both proliferation and differentiation. The expressions of myogenic regulatory transcription factors were also different between satellite cells from different fiber types. MyoD expression, which partially regulates proliferation, was generally expressed at higher levels in p. major satellite cells compared to the b.femoris satellite cells from 33 to 43°C during proliferation and differentiation. Similarly, myogenin expression, which is required for differentiation, was also expressed at higher levels in p. major satellite cells in response to both cold and hot temperatures during proliferation and differentiation than b. femoris satellite cells. These data demonstrate that satellite cells from the anaerobic p. major muscle are more sensitive than satellite cells from the aerobic b. femoris muscle to both hot and cold thermal stress during myogenic proliferation and differentiation.

## Introduction

Posthatch muscle growth occurs through a process called hypertrophy. This process is mediated by a population of adult stem cells termed satellite cells (Smith 1963; Moss and LeBlond 1971; Campion 1984; Hawke and Garry 2001). During the past several years, research has shown that satellite cells are a multipotential mesenchymal stem cell population. As such, satellite cells prefer to follow a myogenic pathway, but may commit to alternative differentiation programs such as osteogenesis or adipogenesis under altered culture conditions (Asakura et al. 2001; Shefer et al. 2004; Vettor et al. 2009).

Satellite cell identity and function are regulated by a number of myogenic regulatory factors (MRF), including myogenic determination factor 1 (MyoD), myogenin (MyoG), and myogenic regulatory factor 4 (MRF4). While MyoD is functionally redundant with another MRF, myogenic factor 5 (Myf5), the expression of at least one of these genes is essential for myoblast proliferation (Rudnicki et al. 1993; Yablonka‐Reuveni and Rivera 1994). Alternately, the function of both MyoG (Brunetti and Goldfine 1990; Yablonka‐Reuveni and Rivera 1994) and MRF4 (Hintenberger et al. 1994; Kassar‐Duchossoy et al. 2004) is to promote differentiation of satellite cells into myotubes.

In broiler chickens, satellite cells are maximally active immediately posthatch and responsive to nutritional regime (Halevy et al. 2000; Mozdziak et al. 2002; Velleman et al. 2010; Kornasio et al. 2011) and environmental changes (Halevy et al. 1998, 2001, 2006; Mozdziak et al. 2002). Satellite cells may respond differently to temperature based upon the fiber type of origin. Satellite cells taken from various fiber types are intrinsically different, as they preferentially differentiate into the same fiber type from which they originated (Feldman and Stockdale 1991; Collins et al. 2005; Huang et al. 2006). Anaerobic type II fibers like the pectoralis major (p. major) muscle contain fast‐twitch fibers providing for rapid movements through glycolytic metabolism and have low levels of blood supply (Rosser et al. 1996; Westerblad et al. 2010). Aerobic type I slow‐twitch fibers have more blood supply and utilize oxidative metabolism for endurance activities (Peter et al. 1972; Dahmane Gošnak et al. 2010). Mixed fiber type muscles, such as the biceps femoris (b. femoris), contain characteristics of both fiber types. Studies comparing chicken satellite cells from type II fast‐twitch anaerobic p. major and mixed fiber type b. femoris, demonstrate that p. major satellite cells are more affected by external factors than b. femoris satellite cells (McFarland et al. 1997; Powell et al. 2014a,b; Harding et al. 2015).

In chickens, satellite cells are maximally active immediately after hatch (Halevy et al. 1998, 2001, 2006; Mozdziak et al. 2002). Therefore, temperature changes that are part of poultry handling during this time may alter the satellite cell activity, thereby affecting muscle growth. The objective of this study was to investigate how temperatures both below and above the normal in vitro temperature of 38°C affects the proliferation and differentiation of chicken satellite cells isolated from different fiber type muscles.

## Materials and Methods

### Isolation of broiler pectoralis major and biceps femoris satellite cells

Satellite cells were previously isolated from the p. major muscle or b. femoris muscle of 5‐week‐old female broilers from a Rock Cornish chicken background and pooled (*gallus domesticus*). Single satellite cells were isolated to create a clonal population using a Quixell cell manipulator robotic system (Stoelting Co., Wood Dale, IL). Clonal populations were expanded, and stored in liquid nitrogen until use (McFarland et al. 1997). This isolation produced a homogenous satellite cell population free of fibroblast and other nonmyogenic cell types.

### Cell culture

Broiler p. major and b. femoris satellite cells were plated simultaneously in 24 well, 0.1% porcine gelatin (Sigma‐Aldrich, St. Louis, MO) coated cell culture plates (Gemini BioProducts, West Sacramento, CA) at 12,000 cells per well for each experimental comparison. Plating was performed with medium consisting of Dulbecco's Modified Eagle Medium (DMEM; Sigma‐Aldrich) with 10% chicken serum, 5% horse serum, 1% antibiotic/antimycotic, and 0.1% gentamicin (Gemini BioProducts). Plates were incubated in a 95% air/5% CO_2_ incubator (Thermo Fisher Scientific, Pittsburgh, PA) at 38°C. Following 24 h for attachment, the medium was changed to feeding medium containing McCoy's 5A (Sigma‐Aldrich) with 10% chicken serum, 5% horse serum, 1% antibiotic/antimycotic, and 0.1% gentamicin (Gemini BioProducts). Plates for the experimental temperatures below or above control (33, 35, 37, 39, 41, or 43°C) were moved to a separate incubator (95% air/5% CO_2_) (Thermo Fisher Scientific) at the desired experimental temperature following attachment of the satellite cells, whereas control cultures remained at 38°C. Once the cultures reached 72 h following attachment (approximately 60–65% confluency), differentiation was induced with a low serum DMEM medium containing 3% horse serum, 1% antibiotic/antimycotic, 0.1% gentamicin (Gemini BioProducts), 0.1% porcine gelatin, and 1 mg/mL bovine serum albumin (Sigma‐Aldrich). Medium was changed every 24 h through 72 h of proliferation and 72 h of differentiation. Digital photomicrographs of the satellite cell cultures were taken every 24 h during proliferation and differentiation using an Olympus IX70 fluorescent microscope (Olympus America, Melville, NY) and QImaging digital camera (QImaging, Burnaby, BC, Canada) equipped with CellSens Imaging software (Olympus America).

### Proliferation assay

Proliferation was assayed through measurement of DNA concentration using Hoechst 33258 fluorochrome as previously described (McFarland et al. 1995). Briefly, every 24 h, medium was removed, one plate was collected and washed with phosphate‐buffered saline (PBS: 137 mmol/L NaCl, 2.68 mmol/L KCl, 1.47 mmol/L KH_2_PO_4_, and 7.81 mmol/L Na_2_HPO_4_, pH 7.08) then stored at −70°C until all plates were collected. Plates were thawed for 15 min at room temperature and treated with 200 *μ*L 0.05% trypsin/ethylenediaminetetraacetic acid (EDTA; Invitrogen) in buffer containing 10 mmol/L Tris, 2 mol/L NaCl, and 1 mmol/L EDTA (TNE) for 7 min and returned to −70°C overnight. Each standard curve and cell culture well was treated with TNE buffer containing 0.2% (1 mg/mL) Hoechst dye (Sigma‐Aldrich) and allowed to shake for 1–2 h. The concentration of DNA labeled with Hoechst dye was measured on a Fluoroskan Ascent FL scanner (Thermo Fisher Scientific, Waltham, MA) and calculated based on a standard curve of double‐stranded calf thymus DNA (Sigma‐Aldrich) run in a plate previously trypsinized with the culture plates. The proliferation assay was plated in two separate experiments with four replicate wells per experiment.

### Differentiation assay

Differentiation was assayed by measuring creatine kinase levels using a modified method of Yun et al. (1997). One plate was collected every 24 h, the medium removed and wells rinsed with PBS, air dried for 15 min, and stored at −70°C until analysis. In brief, plates were thawed for 10–15 min and 500 *μ*L of creatine kinase buffer (20 mmol/L glucose (Thermo Fisher Scientific), 10 mmol/L Mg acetate (Thermo Fisher Scientific), 1.0 mmol/L adenosine diphosphate (Sigma‐Aldrich), 10 mmol/L adenosine monophosphate (Sigma‐Aldrich), 20 mmol/L phosphocreatine (Calbiochem, San Diego, CA), 0.5 U/mL hexokinase (Worthington Biochemical, Lakewood, NJ), 1 U/mL of glucose‐6‐phosphate dehydrogenase (Worthington Biochemical), 0.4 mmol/L thio‐nicotinamide adenine dinucleotide (Oriental Yeast Co., Tokyo, Japan) and 1 mg/mL BSA, Sigma‐Aldrich) was added to each well including the standard curve wells containing creatine phosophokinase at concentrations between 0 and 40 mU/well. The optical density of each well at 405 nm was measured using a BioTek ELx800 (BioTek, Winooski, VT) plate reader. The differentiation assay was plated in two separate experiments with five replicate wells per experiment.

### Myotube width measurements

Digital photomicrographs of the satellite cell cultures taken at 48 h differentiation using an Olympus IX70 fluorescent microscope (Olympus America, Melville, NY) and QImaging digital camera (QImaging, Burnaby, BC, Canada) using CellSens software (Olympus America, Melville, NY) were used to measure myotube widths. The measurement feature of Image Pro Software (Media Cybernetics, Rockville, MD) was applied to 30 randomly selected myotubes for each treatment per experiment from two separately plated experiments. Myotube measurements totaled 60 myotubes widths per temperature and cell type combination. Control myotubes at 38°C were considered separately for each temperature and cell type combination. Myotube measurements were averaged and reported with the standard error of the mean (SEM).

### Real‐time quantitative PCR analysis of gene expression

Both p. major and b. femoris satellite cells were cultured for gene expression analysis as described above at the control (38°C) temperature and variable experimental temperatures (33, 35, 37, 39, 41, or 43°C) and collected every 24 h from 72 h of proliferation through 72 h of differentiation. Plates were removed from the cell culture incubator, media removed, culture wells rinsed with PBS, air dried, and stored at −70°C until analysis. Experiments were plated with a total of six replicate wells per treatment and sampling time. All six replicates from each treatment and sampling time were pooled for RNA extraction. Total RNA was isolated from cell culture plates using RNAzol RT (Molecular Research Center Inc., Cincinnati, OH) according to the manufacturer's protocol. Reverse transcription to produce cDNA was completed using Moloney murine leukemia virus (MMLV) reverse transcriptase reagents (Promega, Madison, WI). Briefly, 1 *μ*g of total RNA was combined with 1 *μ*L 50 mmol/L Oligo dT (Operon, Huntsville, AL) and nuclease‐free water to a volume of 13.5 *μ*L and incubated at 70°C for 5 min, then placed on ice. A mixture of reaction reagents containing 5 *μ*L of 5× MMLV buffer, 1 *μ*L of 10 mmol/L deoxynucleotide triphosphate mix, 0.25 *μ*L of 40 U/*μ*L RNAsin, 1 *μ*L of 200 U/*μ*L MMLV reverse transcriptase, and nuclease‐free water up to 11.5 *μ*L per reaction were added to each tube. Samples were then incubated at 55°C for 60 min and 90°C for 10 min. Following incubation 25 *μ*L of nuclease‐free water was added to each reaction. Real‐time quantitative PCR (RT‐qPCR) was performed using DyNAmo Hot Start SYBR green qPCR master mix (Finnzymes, Ipswich, MA) according to manufacturer's instructions for analysis of glyceraldehyde‐3‐phosphate dehydrogenase (GAPDH), myogenic differentiation 1 (MyoD), myogenin (MyoG), and myogenic regulatory factor 4 (MRF4) expression. Primer sequences and GenBank accession numbers are listed in Table 1. Primer specificities were confirmed by DNA sequencing of gel purified PCR products (Molecular and Cellular Imaging Center, The Ohio Agricultural Research and Development Center, Wooster). In brief, 2 *μ*L of cDNA was combined with a reaction mix containing 10 *μ*L 2× DyNAmo HS SYBR green master mix, 1 *μ*L of primer mix containing 10 μmol/L each forward and reverse primers, and 7 *μ*L of nuclease‐free water. Reactions were run on a DNA Engine Opticon 2 real‐time machine (BioRad, Hercules, CA) with the following cycling conditions: 95°C for 15 min, 34 cycles of 94°C for 30 sec, 55°C for 30 sec, and 72°C for 30 sec, followed by a final extension at 72°C for 5 min. Amplification specificity was confirmed by resolving randomly selected samples from all RT‐qPCR reactions on a 1% agarose gel. Six serial dilutions of purified PCR products were used to produce standard curves for each gene. Serial dilutions were assigned arbitrary concentrations between 1 and 1 × 10^6^. The arbitrary values of samples were calculated based on the relation of curve Ct values to assigned standard curve concentrations. Data were then normalized to the average GAPDH expression of pooled samples from all 38°C treatments by dividing the arbitrary molar concentration of the samples by the arbitrary GAPDH molar value for each cell type at each sampling time as GAPDH expression was affected by temperature. Two experiments were individually plated with three identical technical replicate RT‐qPCR reactions run per sample for each gene per experiment. Data from a single experiment that best demonstrated the observed trends in the total data were selected as a representative of both experiments analyzed.

**Table 1:** Primer sequences for real time quantitative PCR

Primer	Sequence^a^	Coding region	Product size	Annealing temperature
GAPDH	5′‐GAG GGT AGT GAA GGC TGC TG‐3′ (forward)	504–523	200 bp^b^	55°C
	5′‐CCA CAA CAC GGT TGC TGT AT‐3′ (reverse)	684–703		55°C
MyoD	5′‐GAC GGC ATG ATG GAG TAC AG‐3′ (forward)	553–572	201 bp	58°C
	5′‐AGC TTC AGC TGG AGG CAG TA‐3′ (reverse)	734–753		58°C
MyoG	5′‐CCT TTC CCA CTC CTC TCC AAA‐3′ (forward)	813–833	175 bp	58°C
	5′‐GAC CTT GGT CGA AGA GCA ACT‐3′ (reverse)	967–987		58°C
MRF4	5′‐AGG CTC TGA AAA GGA GGA CTG T ‐3′ (forward)	341–362	307 bp	58°C
	5′‐AGG CTG CTG GAA GCC GAC GAC T ‐3′ (reverse)	626–647		58°C

a Primer sequences were designed from the following GenBank accession numbers: glyceraldehyde‐3‐phosphate dehydrogenase (GAPDH), U94327.1; myogenic differentiation 1 (MyoD), AY641567.1; Myogenin (MyoG), AY560111.3; Myogenic regulatory factor 4 (MRF4) GU223068.

b Base pairs of DNA.

### Statistical analysis

For gene expression, two experiments were plated with three technical replicate RT‐qPCR reactions run per sample for each gene per experiment. Statistical analysis was performed on all experiments and data from a single experiment were selected as representative of both experiments analyzed. Datasets were regressed across incubation temperature within each sampling time in the MIXED procedure of SAS (SAS Institute Inc 2011). Appropriate linear contrast statements were used to obtain the linear equation for each cell type across the experimental temperatures. An additional contrast statement was used to test for sensitivity differences between the linear responses across temperature and between the cell types. The least square means (lsmeans) statement was used to determine means and standard error of the mean. The model included the main effects of incubation temperature, cell type, and their interaction. Simultaneously, the data were sliced by temperature to signify differences between cell types within each experimental temperature. An additional analysis was completed for the extreme temperatures (33°C and 43°C) compared to the respective control temperature to understand how gene expression changed over time. A one‐way ANOVA completed within each temperature and cell type combination was used to determine differences across time, therefore, the model included the main effect of time. The lsmeans statement was used to determine means and standard error of the mean. The pdiff option was used to separate interaction means. Differences were considered significant at *P *<* *0.05.

For proliferation and differentiation, data were analyzed as an incomplete block design, as each experimental temperature was paired with the 38°C control. Statistical analysis was performed on all experiments and data from a single experiment were selected as representative of both experiments analyzed. The lsmeans statement was used to determine means and standard errors of the means. The MIXED procedure of SAS was used to determine differences between cell types within a specific temperature and sampling time. The SLICE option was used to separate interaction means. The REG procedure of SAS was used to evaluate the linear response across temperature for each cell type. Contrast statements in the GLM procedure of SAS were used to determine response differences between cell types. Differences were considered significant at *P *<* *0.05.

For myotube width analysis, data were analyzed as an incomplete block design, as each experimental temperature was paired with the 38°C control. The lsmeans statement was used to determine means and standard errors of the means. The GLIMMIX procedure of SAS was used to determine differences between cell types within a specific temperature and sampling time. The main effects included temperature, cell line, and the interaction. The SLICE option was used to separate interaction means. A second analysis was also completed in GLIMMIX procedure of SAS to determine differences between temperatures within each cell line. Two separate analyses were conducted on each cell type with the main effect of temperature. Differences were considered significant at *P *<* *0.05.

## Results

### Effect of temperature on satellite cell proliferation

**Figure 1:**
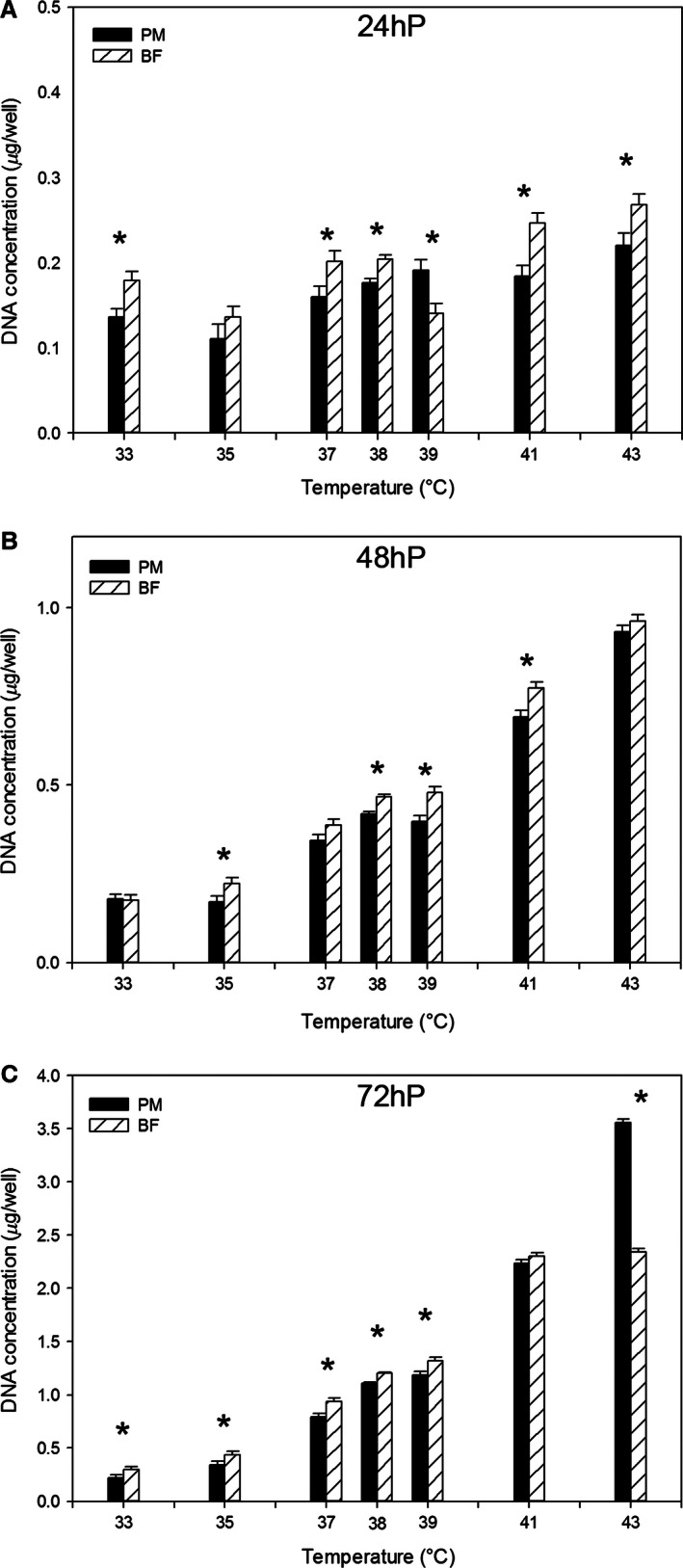
The effect of temperature on satellite cell proliferation. Proliferation was measured in pectoralis major (PM) and biceps femoris (BF) satellite cells cultured at temperatures below (33, 35, 37°C) or above (39, 41, 43°C) control (38°C) at (A) 24 h of proliferation (24hP) (B) 48 h of proliferation (48hP), or (C) 72 h of proliferation (72hP). Bars represent standard error of the mean. Data with * indicate significant difference between cell types (*P *≤* *0.05).

Proliferation of p. major and b. femoris cells was measured at 24, 48, and 72 h of proliferation when cultured at temperatures below (33, 35, or 37°C) or above (39, 41, or 43°C) control (38°C). At 24 h of proliferation, p. major satellite cells had significantly reduced proliferation compared to the b. femoris satellite cells at 33, 37, 38, 41, and 43°C. At 39°C, p. major proliferation was significantly greater than b. femoris. There was no significant difference between the cell types at 35°C (Fig. 1A). Linear regression demonstrated that proliferation of both p. major (slope: 0.009 ± 0.001) and b. femoris (slope: 0.009 ± 0.002) satellite cells increased across temperature from 33 to 43°C, but there was no difference in the rate of increase between the cell types at 24 h of proliferation. Comparing cell types at 48 h, proliferation was significantly lower in p. major satellite cells at 35, 38, 39, and 41°C compared to b. femoris satellite cells. There was no difference in proliferation between cell types at 33, 37, or 43°C (Fig. 1B). The proliferation of both p. major (slope: 0.073 ± 0.004) and b. femoris (slope: 0.078 ± 0.003) satellite cells linearly increased with temperature, with no difference in the rate of increase. By 72 h of proliferation, satellite cells of both cell types cultured below 38°C (33, 35, and 37°C) exhibited reduced proliferation compared to 38°C, whereas satellite cells cultured above 38°C (39, 41, and 43°C) exhibited greater proliferation. Proliferation of p. major satellite cells was significantly less than that of b. femoris satellite cells between 33 and 39°C at 72 h of proliferation. The proliferation of the two cell types was equivalent at 41°C and at 43°C p. major satellite cell proliferation was significantly higher compared to the b. femoris satellite cells (Fig. 1C). The proliferation of both cell types linearly increased from 33 to 43°C, but the rate of increase was greater in the p. major satellite cells (slope: 0.308 ± 0.018) than the b. femoris satellite cells (slope: 0.225 ± 0.009) at 72 h.

**Figure 2:**
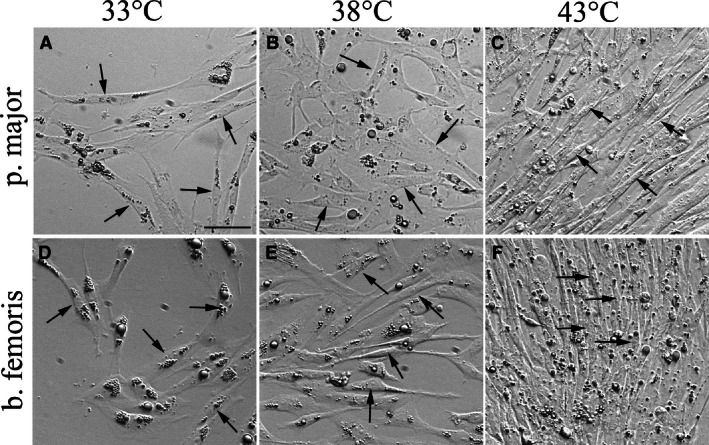
The effect of temperature on pectoralis major (p. major) and biceps femoris (b. femoris) satellite cell growth. Photomicrographs at 72 h of proliferation of p. major (A–C) and b. femoris (D–F) satellite cells grown at 33°C (A, D), 38°C (B, E), and 43°C (C, F). Arrows highlight the satellite cells. The unsharp mask of Photoshop was applied equally to all images to improve clarity of cells and brightness was adjusted to a similar level in all pictures. Scale bar = 50 *μ*m.

Temperature‐induced differences in proliferation produced morphological differences in both p. major (Fig. 2A–C) and b. femoris (Fig. 2D–F) satellite cell cultures. Compared to the control 38°C cultures, the 33°C cultures of both cell types were more sparsely populated with satellite cells (Fig. 2A, D, B, E, arrows), indicative of reduced proliferation of the satellite cells when incubated at 33°C. In contrast, the p. major and b. femoris satellite cells at 43°C (Fig. 2C, F) were densely packed and exhibited alignment of the satellite cells compared to the 38°C cultures (Fig. 2B, E).

Pectoralis major satellite cells cultured at 33°C proliferated significantly less than p. major satellite cells at 38°C at all sampling times (Table 2). In contrast, b. femoris satellite cells cultured at 33 and 38°C were not significantly different until 48 and 72 h when the proliferation was lower in the 33°C cultures. Proliferation of the p. major satellite cells significantly increased from 0 h through 72 h of proliferation at both 33 and 38°C. This was also true of b. femoris satellite cells cultured at 38°C. Biceps femoris satellite cell proliferation increased significantly at 33°C, except between 24 and 48 h when the level of proliferation remained constant.

**Table 2:** Effect of temperature on proliferation^1^ at 38 and 33°C

Time	PM 38°C	PM 33°C	BF 38°C	BF 33°C
0hP	0.09 ± 0.004^a^^,^ z	0.07 ± 0.009^b^^,^ z	0.08 ± 0.004^ab^^,^ z	0.08 ± 0.009^ab^^,^ z
24hP	0.18 ± 0.005^b^^,^ y	0.14 ± 0.010^c^^,^ y	0.20 ± 0.005^a^^,^ y	0.18 ± 0.010^ab^^,^ y
48hP	0.42 ± 0.007^b^^,^ x	0.18 ± 0.01^c^^,^ x	0.47 ± 0.007^a^^,^ x	0.18 ± 0.014^c^^,^ y
72hP	1.11 ± 0.013^b^^,^ w	0.22 ± 0.027^d,^ w	1.20 ± 0.013^a^^,^ w	0.296 ± 0.027^c^^,^ x

PM, pectoralis major; BF, biceps femoris.

^1^Proliferation was quantified through mean DNA content (*μ*g per well) ±SEM.

^a‐c^Means across cell type and temperature at each sampling time; values without common letters are different (*P *<* *0.05).

^w‐z^Means across sampling times for each cell type and temperature combination; values without common letters are different (*P *<* *0.05).

At 43°C, temperature did not affect proliferation in either cell type at 0 h (Table 3). However, from 24 through 72 h, proliferation was significantly higher in both the p. major and b. femoris satellite cells cultured at 43°C compared to the cells incubated at 38°C. Increased proliferation due to temperature was greatest in the p. major satellite cells at 72 h, when the proliferation of the 43°C p. major satellite cells was significantly higher than both the p. major satellite cells grown at 38°C and the b. femoris satellite cells at 38 and 43°C.

**Table 3:** Effect of temperature on proliferation^1^ at 38 and 43°C

Time	PM 38°C	PM 43°C	BF 38°C	BF 43°C
0hP	0.09 ± 0.004^a^^,^ z	0.09 ± 0.012^a^^,^ z	0.08 ± 0.004^a^^,^ z	0.08 ± 0.012^a^^,^ z
24hP	0.18 ± 0.005^c^^,^ z	0.22 ± 0.014^b^^,^ y	0.20 ± 0.005^bc^^,^ y	0.27 ± 0.012^a^^,^ y
48hP	0.42 ± 0.007^b^^,^ y	0.93 ± 0.017^a^^,^ x	0.47 ± 0.007^b^^,^ x	0.96 ± 0.017^a^^,^ x
72hP	1.11 ± 0.013^c^^,^ x	3.56 ± 0.034^a^^,^ w	1.20 ± 0.013^c^^,^ w	2.34 ± 0.034^b^^,^ w

PM, pectoralis major; BF, biceps femoris.

^1^Proliferation was quantified through mean DNA content (*μ*g per well) ±SEM.

^a‐c^Means across cell type and temperature at each sampling time; values without common letters are different (*P *<* *0.05).

^w‐z^Means across sampling times for each cell type and temperature combination; values without common letters are different (*P *<* *0.05).

### Effect of temperature on satellite cell differentiation

**Figure 3:**
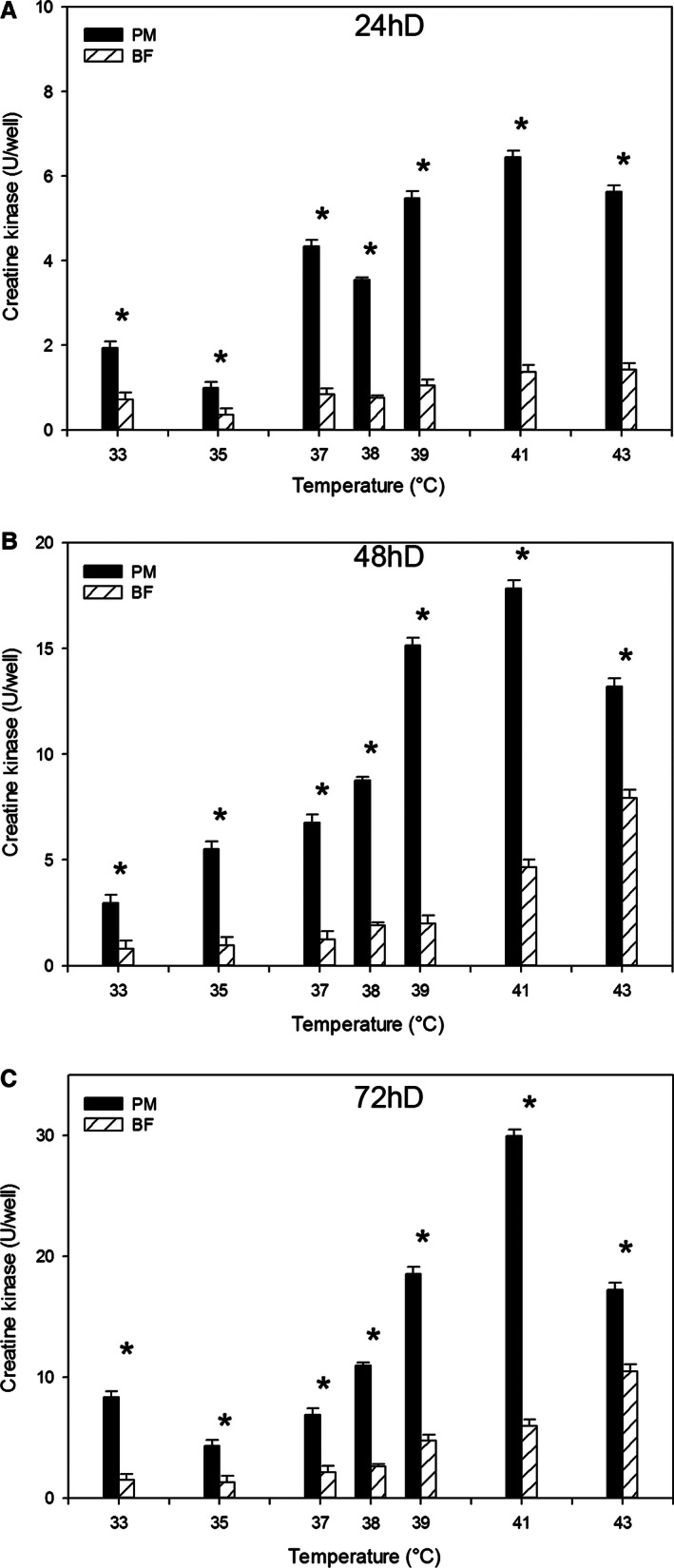
The effect of temperature on satellite cell differentiation. Differentiation was measured in pectoralis major (PM) and biceps femoris (BF) satellite cells cultured at temperatures below (33, 35, 37°C) or above (39, 41, 43°C) control (38°C) at (A) 24 h of differentiation (24hD), (B) 48 h of differentiation (48hD), or (C) 72 h of differentiation (72hD). Bars represent standard error of the mean. Data with * indicate significant difference between cell types (*P *≤* *0.05).

**Figure 4:**
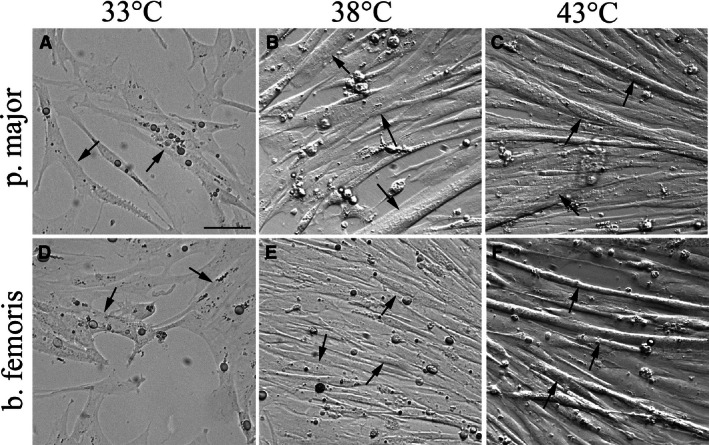
The effect of temperature on pectoralis major (p. major) and biceps femoris (b. femoris) satellite cell myotube formation. Photomicrographs at 72 h of differentiation of p. major (A–C) and b. femoris (D–F) satellite cells grown at 33°C (A, D), 38°C (B, E), and 43°C (C, F). Arrows indicate satellite cell‐derived myotubes. The unsharp mask of Photoshop was applied equally to all images to improve clarity of cells and brightness was adjusted to a similar level in all pictures. Scale bar = 50 *μ*m.

At 24, 48, and 72 h of differentiation, p. major satellite cell differentiation was greater (*P *<* *0.003) than that of b. femoris at all temperatures from 33 to 43°C (Fig. 3A–C). At each sampling time, both the p. major (slope 24 h of differentiation (D): 0.51 ± 0.046; 48hD: 1.38 ± 0.127; 72hD: 1.85 ± 0.264) and b. femoris (slope 24hD: 0.10 ± 0.010; 48hD: 0.68 ± 0.058; 72hD: 0.85 ± 0.082) satellite cells demonstrated a linear increase (*P *<* *0.001) in differentiation with increased temperature. At all three sampling times, the increase in differentiation of the p. major satellite cells was greater than that of the b. femoris. At 72 h of differentiation, limited myotube formation was observed at 33°C for both the p. major (Fig. 4A, arrows) and b. femoris (Fig. 4D, arrows) cultures compared to the control at 38°C (Fig. 4B, E, respectively). In contrast, both the p. major and b. femoris cells grown at 43°C generated densely packed and aligned myotubes (Fig. 4C, F, arrows, respectively), compared to both the 33°C (Fig. 4A, D) and 38°C (Fig. 4B, E) cultures.

Satellite cells from the p. major muscle incubated at 33°C had increased differentiation compared to control 38°C p. major satellite cells at 0 h, but decreased differentiation compared to the control cells at 24, 48, and 72 h (Table 4). There was no difference in differentiation between b. femoris satellite cells grown at 33°C and 38°C at 0, 24, or 48 h. At 72 h, b. femoris satellite cells at 33°C had reduced differentiation compared to the 38°C control cells. Across time, p. major satellite cells grown at 38°C had increased differentiation from 0 to 72 h. Differentiation of p. major satellite cells at 33°C did not increase between 0 and 24 h, but increased from 24 to 72 h. Biceps femoris satellite cells grown at 38°C were equivalent between 0 and 24 h, but differentiation was increased from 24 through 72 h. Differentiation of b. femoris satellite cells grown at 33°C increased between 0 and 24 h, as well as 48 and 72 h, but did not increase between 24 and 48 h.

**Table 4:** Effect of temperature on differentiation^1^ at 38 and 33°C

Time	PM 38°C	PM 33°C	BF 38°C	BF 33°C
0hD	0.87 ± 0.014^b^^,^ z	1.02 ± 0.033^a^^,^ z	0.55 ± 0.015^c^^,^ z	0.49 ± 0.038^c^^,^ z
24hD	3.55 ± 0.060^a^^,^ y	1.94 ± 0.144^b^^,^ z	0.76 ± 0.061^c^^,^ z	0.72 ± 0.161^c^^,^ y
48hD	8.76 ± 0.161^a^^,^ x	2.97 ± 0.380^b^^,^ y	1.89 ± 0.16^bc^^,^ y	0.79 ± 0.380^c^^,^ y
72hD	11.0 ± 0.224^a^^,^ w	8.32 ± 0.500^b^^,^ x	2.58 ± 0.211^c^^,^ x	1.49 ± 0.500^d,^ x

PM, pectoralis major; BF, biceps femoris.

^1^Differentiation was quantified through mean units of creatine kinase ±SEM.

^a‐c^Means across cell type and temperature at each sampling time; values without common letters are different (*P *<* *0.05).

^w‐z^Means across sampling times for each cell type and temperature combination; values without common letters are different (*P *<* *0.05).

The p. major satellite cells had higher levels of differentiation at both 43 and 38°C compared to the b. femoris satellite cells at the same temperature at 0, 24, and 48 h of differentiation (Table 5). Within cell type, there was no difference in the differentiation of p. major satellite cells grown at 38 and 43°C at 0 h. From 24 through 72 h, the p. major satellite cells incubated at 43°C had greater differentiation than p. major satellite cells grown at 38°C. There was no difference between b. femoris satellite cells at 38 or 43°C at 0 h, but from 24 to 72 h, differentiation was significantly increased in b. femoris cells at 43°C compared to the b. femoris cells incubated at the control temperature of 38°C. Across time, the differentiation of p. major satellite cells grown at both 38 and 43°C increased significantly from 0 to 72 h of differentiation. The b. femoris satellite cell differentiation at 38°C increased only between 24 and 48 h. There was no change in differentiation between 0 and 24 h or 48 and 72 h, respectively. In b. femoris satellite cells incubated at 43°C, differentiation was unchanged between 0 and 24 h, but increased from 24 to 72 h.

**Table 5:** Effect of temperature on differentiation^1^ at 38 and 43°C

Time	PM 38°C	PM 43°C	BF 38°C	BF 43°C
0hD	0.87 ± 0.014^a^^,^ z	0.87 ± 0.034^a^^,^ z	0.55 ± 0.015^b^^,^ z	0.60 ± 0.034^b^^,^ z
24hD	3.55 ± 0.060^b^^,^ y	5.64 ± 0.144^a^^,^ y	0.76 ± 0.061^d,^ y	1.43 ± 0.144^c^^,^ y
48hD	8.76 ± 0.161^b^^,^ x	13.20 ± 0.380^a^^,^ x	1.89 ± 0.163^c^^,^ y	7.93 ± 0.380^b^^,^ y
72hD	11.0 ± 0.224^b^^,^ w	17.23 ± 0.560^a^^,^ w	2.58 ± 0.211^c^^,^ y	10.49 ± 0.56^b^^,^ x

PM, pectoralis major; BF, biceps femoris.

^1^Differentiation was quantified through mean units of creatine kinase ±SEM.

^a‐c^Means across cell type and temperature at each sampling time; values without common letters are different (*P *<* *0.05).

^w‐z^Means across sampling times for each cell type and temperature combination; values without common letters are different (*P *<* *0.05).

Myotube width at 48 h of differentiation was measured. The p. major satellite cells at 35°C had a myotube width of 12.4 ± 1.0 *μ*m that was significantly narrower compared to the control (38°C: 13.8 ± 0.77 *μ*m). In the 43°C cultures, p. major myotube width, 17.9 ± 1.0 *μ*m, was wider compared to the p. major myotube width at 38°C (13.8 ± 0.77 *μ*m). Temperatures other than 35 and 43°C did not result in myotube width differences (33°C: 13.2 ± 1.0 *μ*m; 37°C: 13.6 ± 1.0 *μ*m; 39°C: 13.0 ± 1.0 *μ*m; 41°C: 12.9 ± 1.0 *μ*m), compared to the average of control 38°C width of 13.8 ± 0.77 *μ*m. Biceps femoris‐derived myotube widths at any of the experimental temperatures (33°C: 13.7 ± 0.95 *μ*m; 35°C: 14.4 ± 0.95 *μ*m; 37°C: 12.4 ± 0.95 *μ*m; 39°C: 13.0 ± 0.95 *μ*m; 41°C: 12.7 ± 0.95 *μ*m; 43°C: 14.1 ± 0.95 *μ*m) were not significantly different compared to the b. femoris myotube width at the control temperature (38°C: 13.5 ± 0.72 *μ*m).

### Effect of temperature on myogenic gene expression

**Figure 5:**
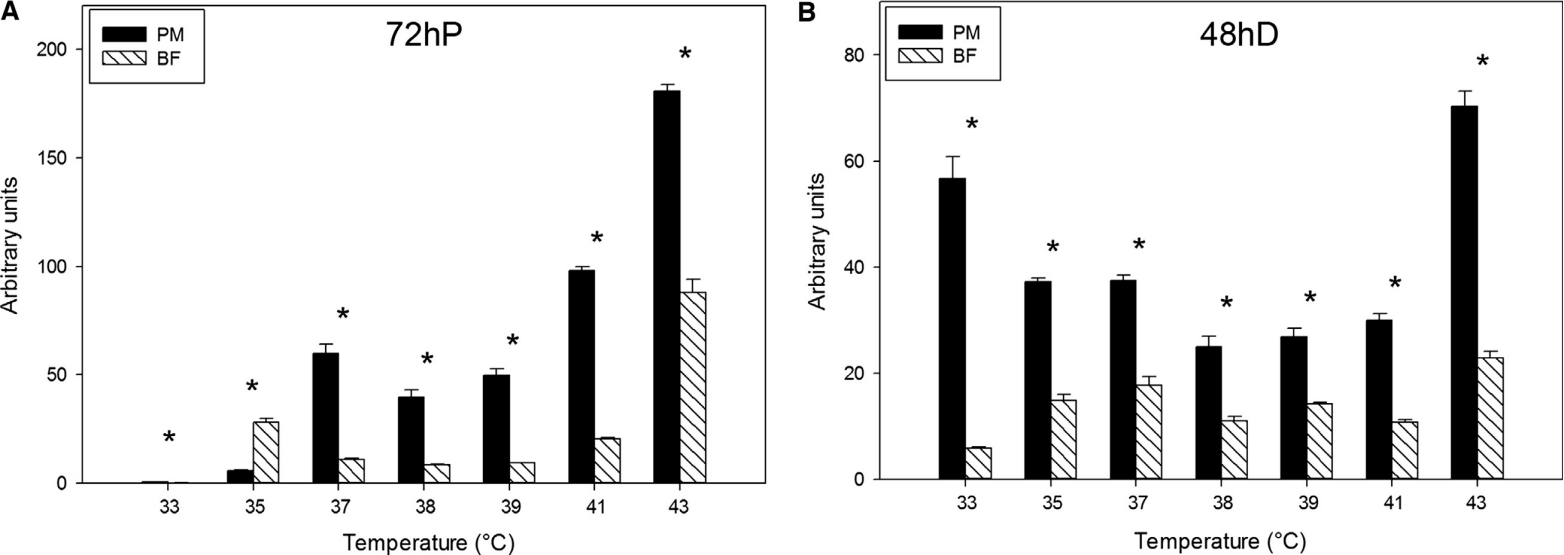
Expression of myogenic gene myogenic differentiation factor 1 (MyoD). The expression of MyoD at (A) 72 h of proliferation (72hP) and (B) 48 h of differentiation (48hD) in pectoralis major (PM) and biceps femoris (BF) satellite cells grown at temperatures between 33 and 43°C. Bars represent standard error of the mean. Data with * indicate significant difference (*P *≤* *0.05) between cell fiber types at individual temperatures.

The expression of myogenic regulatory factors MyoD, MyoG, and MRF4 was measured. Expression of MyoD at 72 h of proliferation was higher in p. major satellite cells than b. femoris satellite cells at all temperatures except 35°C, when b. femoris expressed MyoD at a greater level (Fig. 5A). Linear regression analysis of these data across temperatures from 33 to 43°C demonstrated that both p. major (slope: 15.65) and b. femoris (slope: 4.60) increased linearly with increasing temperature, but p. major satellite cells increased MyoD expression at a greater rate than b. femoris satellite cells. At 48 h of differentiation, p. major satellite cell expression of MyoD was significantly higher than that of the b. femoris satellite cells at all temperatures (Fig. 5B). At 48 h of differentiation p. major satellite cells did not have a linear increase in MyoD expression across temperature, whereas the b. femoris satellite cells increased (slope: 0.99) linearly.

**Figure 6:**
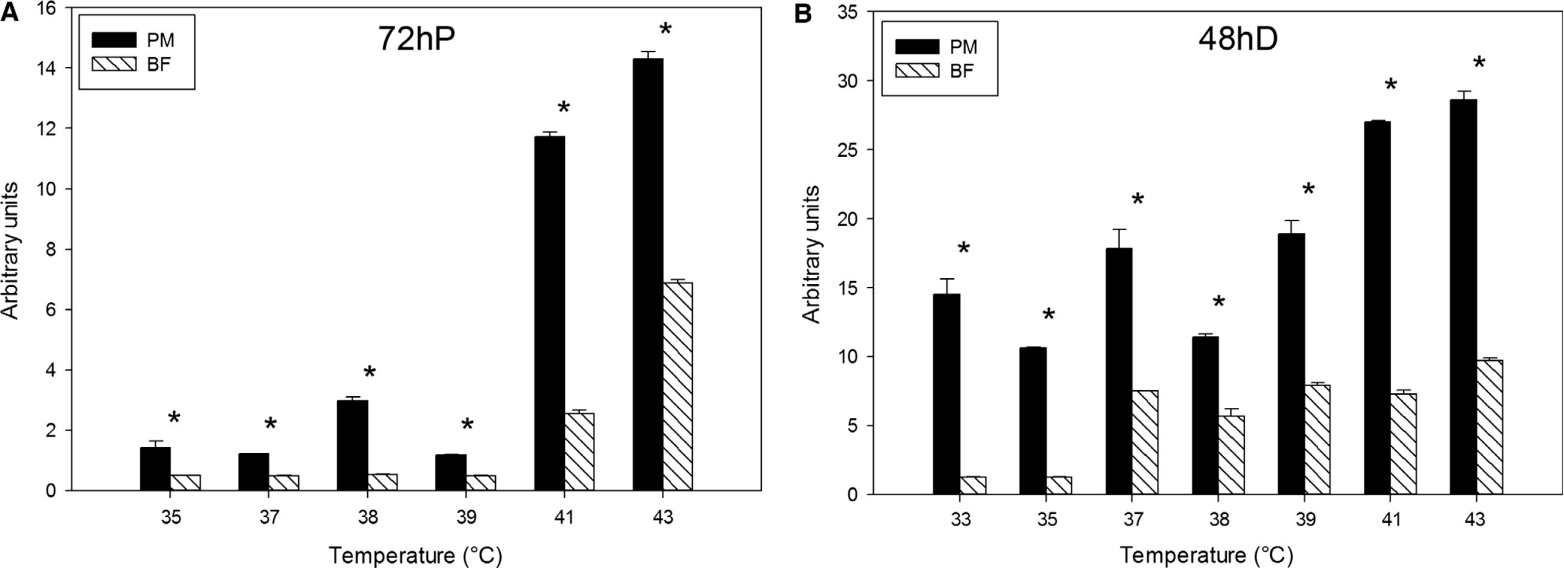
Expression of myogenic regulatory transcription factor myogenin (MyoG). The expression of MyoG at (A) 72 h of proliferation (72hP) and (B) 48 h of differentiation (48hD) in pectoralis major (PM) and biceps femoris (BF) satellite cells grown at temperatures between 33 and 43°C. Bars represent standard error of the mean. Data with * indicate significant difference (*P *≤* *0.05) between cell fiber types at individual temperatures.

Expression of MyoG at 72 h of proliferation (Fig. 6A) and 48 h of differentiation (Fig. 6B) was greater in the p. major satellite cells than the b. femoris satellite cells at all temperatures. Both p. major and b. femoris satellite cell MyoG expression increased linearly (*P *<* *0.001) across temperatures from 33 to 43°C. The p. major (slope: 1.84) increase in MyoG expression across temperature at 72 h of proliferation was greater (*P *<* *0.001) than that of the b. femoris (slope: 0.76) increase. Both the p. major and b. femoris cell types also increased MyoG expression linearly (*P *<* *0.001) at 48 h of differentiation, with the p. major (slope: 1.68) satellite cells having a greater increase in MyoG expression (*P *<* *0.001) compared to the b. femoris (slope: 0.87) satellite cells.

**Figure 7:**
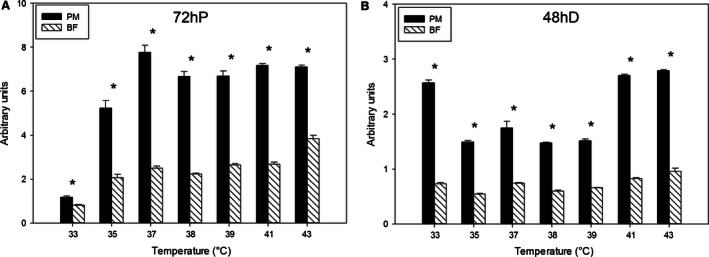
Expression of myogenic regulatory transcription factor myogenin (MRF4). The expression of MRF4 at (A) 72 h of proliferation (72hP) and (B) 48 h of differentiation (48hD) in pectoralis major (PM) and biceps femoris (BF) satellite cells grown at temperatures between 33 and 43°C. Bars represent standard error of the mean. Data with * indicate significant difference (*P *≤* *0.05) between cell fiber types at individual temperatures.

At 72 h of proliferation (Fig. 7A) and 48 h of differentiation (Fig. 7B), MRF4 expression in the p. major satellite cells was greater than that of the b. femoris satellite cells at all temperatures. The expression of MRF4 in p. major (slope: 0.58) satellite cells increased linearly (*P *<* *0.001) at 72 h of proliferation as temperature increased, whereas the b. femoris expression of MRF4 did not have a linear trend (*P *=* *0.06). By 48 h of differentiation, the expression of both p. major (slope: 0.053) and b. femoris (0.027) satellite cells increased linearly (*P *<* *0.001) with increased temperature from 33 to 43°C. The p. major satellite cells had a greater (*P *<* *0.001) increase in MRF4 expression than the b. femoris satellite cells.

## Discussion

Satellite cells are maximally active immediately posthatch and are highly responsive to temperature variation, which affects proliferation, differentiation, and cellular fate of satellite cells (Halevy et al. 2000; Velleman et al. 2010; Clark et al. 2016). Clark et al. (2016) demonstrated increased proliferation, differentiation, and myotube width of in vitro turkey p.major satellite cells in response to rising culture temperatures up to 43°C. Furthermore, Yamaguchi et al. (2010) reported that a mild continuous thermal challenge of 39°C on cultured SC during proliferation and differentiation increased myotube size. This study identified minimal changes to myotube width, with only the p. major satellite cells producing larger myotubes at 43°C. Yamaguchi et al. (2010) observed that, temperatures above 39°C resulted in poorly formed myotubes. These variations in myotube formation in response to high temperature may be due to species differences, as Yamaguchi et al. (2010) isolated satellite cells from humans and mice, which have a lower internal body temperature than chickens and turkeys (Aiello 2015). Although some information is available on the effect of temperature on myogenic satellite cells, little is known about how temperature affects satellite cells from different muscle fiber types.

The p. major muscle is comprised of fast‐twitch glycolytic type IIb fibers and the b. femoris is a mixed muscle containing slow‐twitch aerobic type I fibers, and type IIA and IIB fibers (Rosser et al. 1996; Dahmane Gošnak et al. 2010; Westerblad et al. 2010). Satellite cells isolated from the p. major and b. femoris muscles in broilers vary in growth characteristics and response to mitogen‐like growth factors, with the p. major satellite cells generally being more sensitive to changes than the b. femoris satellite cells (McFarland et al. 1997, 2007; Kocamis et al. 2001). For example, p. major satellite cells are more sensitive to feed restriction than b. femoris satellite cells (Powell et al. 2014a). McFarland et al. (2007) showed p. major satellite cells in vitro were more sensitive to the proliferative inhibitory effects of myostatin than b. femoris satellite cells. Given the sensitivity of p. major satellite cells to external changes, it was not surprising that proliferation of p. major satellite cells were more sensitive to culture temperatures compared to b. femoris satellite cells. Additionally, the expression of MyoD, a transcription factor that promotes satellite cell proliferation (Rudnicki et al. 1993; Yablonka‐Reuveni and Rivera 1994), was generally expressed at greater levels in p. major satellite cells compared to b. femoris satellite cells. The change in the expression levels of MyoD across temperature were also increased in the p. major satellite cells compared to the b. femoris satellite cells. The increased proliferation and MyoD expression with higher temperatures were consistent with previous research in turkey p. major satellite cells (Clark et al. 2016).

Pectoralis major and b. femoris satellite cells were also shown by McFarland et al. (1997) to vary in differentiation properties. In low serum conditions, the p. major satellite cells differentiated into myofibers faster than the b. femoris satellite cells. This trend was also observed in this study, as p. major satellite cell differentiation was greater than b. femoris satellite cell differentiation under all experimental conditions, and was affected more by increasing temperatures. These results are similar to those obtained by Clark et al. (2016), who observed increased differentiation of turkey satellite cells with higher temperatures. The increased differentiation of the p. major satellite cells was further supported by the expression of the myogenic regulatory transcription factors MyoG and MRF4. The expression of both genes was higher in p. major satellite cells compared to the b. femoris satellite cells during differentiation. Additionally, temperature had a greater effect on gene expression in the p. major satellite cells compared to b. femoris satellite cells. Taken together, these results suggest that the p. major satellite cells are more sensitive to temperature changes compared to b. femoris satellite cells.

The differences demonstrated between the p. major and b. femoris satellite cells by this and other reports (McFarland et al. 1997; Powell et al. 2014a,b; Harding et al. 2015) are likely due to differences in metabolic properties of the originating muscle fiber. The b. femoris is a mixed muscle type containing both type I and type II muscle fibers, and is a predominantly aerobic muscle. Aerobic fiber types preferentially utilize glucose and thereby efficiently produce large amounts of energy for growth and differentiation. Alternately, p. major muscle is predominantly comprised of type IIB fast‐twitch anaerobic fibers. Anaerobic metabolism occurs through lactic acid fermentation, a much less efficient form of energy production than aerobic metabolism (Westerblad et al. 2010). The more efficient aerobic energy production of the mixed fiber type b. femoris satellite cells may provide these cells with greater energy for maintaining cellular homeostasis. A limited availability of energy for the p. major satellite cells to maintain normal function would result in their greater sensitivity to external stimuli.

This study furthers our understanding of the effect of temperature on myogenic satellite cells isolated from different fiber type muscles. The p. major muscle satellite cells are more sensitive to temperature change in vitro than b. femoris satellite cells, making it likely that the p. major satellite cells are also more sensitive in vivo. Since the p. major muscle (breast muscle), is the largest muscle by weight and sought out as a low‐fat meat choice, it is the most economically valuable part of the bird. As such, it is essential that poultry producers take into account the effect of temperature on resulting meat quality.

## Conflict of Interest

None declared.
